# SnSe_2_ Quantum Dots: Facile Fabrication and Application in Highly Responsive UV-Detectors

**DOI:** 10.3390/nano9091324

**Published:** 2019-09-15

**Authors:** Xiangyang Li, Ling Li, Huancheng Zhao, Shuangchen Ruan, Wenfei Zhang, Peiguang Yan, Zhenhua Sun, Huawei Liang, Keyu Tao

**Affiliations:** Shenzhen Key Laboratory of Laser Engineering, College of Physics and Optoelectronic Engineering, Shenzhen University, Shenzhen 518060, China; 2170285209@email.szu.edu.cn (X.L.); 1800281011@email.szu.edu.cn (H.Z.); scruan@szu.edu.cn (S.R.); zhangwf@szu.edu.cn (W.Z.); yanpg@szu.edu.cn (P.Y.); szh@szu.edu.cn (Z.S.); hwliang@szu.edu.cn (H.L.); taokeyu@szu.edu.cn (K.T.)

**Keywords:** SnSe_2_ quantum dots, graphene, phototransistor, UV-detector

## Abstract

Synthesizing quantum dots (QDs) using simple methods and utilizing them in optoelectronic devices are active areas of research. In this paper, we fabricated SnSe_2_ QDs via sonication and a laser ablation process. Deionized water was used as a solvent, and there were no organic chemicals introduced in the process. It was a facile and environmentally-friendly method. We demonstrated an ultraviolet (UV)-detector based on monolayer graphene and SnSe_2_ QDs. The photoresponsivity of the detector was up to 7.5 × 10^6^ mAW^−1^, and the photoresponse time was ~0.31 s. The n–n heterostructures between monolayer graphene and SnSe_2_ QDs improved the light absorption and the transportation of photocarriers, which could greatly increase the photoresponsivity of the device.

## 1. Introduction

Graphene-based electronic and optoelectronic devices have attracted extensive attention [1,2,3]. Mueller et al. demonstrated a vertical incidence metal–graphene–metal photodetector with an external responsivity of 6.1 mAW^−1^ at 1.55 µm [4]. The photoresponsivity was limited by the low absorption of the graphene. Quantum dots (QDs) can break this limitation. They can act as light absorption spots. The photo-induced carriers in them can transfer into the graphene film, and the charges in the graphene film transport to the electrodes quickly. Thus, the responsivity of a graphene-based device is improved [5,6,7]. Cheng et al. showed a phototransistor based on graphene and graphene QDs with a photoresponsivity of up to 4 × 10^10^ mAW^−1^, but the response time was 10 s [8]. Sun et al. constructed an infrared photodetector based on graphene and PbS QDs with a responsivity of up to 10^10^ mAW^−1^ and a response time of 0.26 s [9]. Sun et al. demonstrated a UV phototransistor based on graphene and ZnSe/ZnS core/shell QDs. Its responsivity was up to 10^6^ mAW^−1^ and the response time was 0.52 s [10].

In order to fabricate the QD solution with uniform distribution, the wet chemical method was commonly used. Some organic solvents, such as toluene or pyridine, were used in the process [9,10]. The chemical groups can cap the surface of the QDs and modify their charge transfer property, thus influencing the photo responsivity of the device. Synthesizing QDs using facile and green methods and utilizing them in optoelectronic devices are active areas of research.

Two-dimensional transition-metal dichalcogenides (TMDCs) have been applied in fluorescent imaging [11], biological sensing [12], and photocatalytic [13] due to their unique optoelectronic properties. Tin diselenide (SnSe_2_) is a semiconductor in the TMDCs family. SnSe_2_ QDs can be used in fast and highly responsive phototransistors since they have a tunable bandgap and high quantum efficiency. In this paper, SnSe_2_ QDs were fabricated via sonication and a laser ablation process. The deionized water was used as a solvent, and there were no organic chemicals introduced in the process. It was a facile and environmentally-friendly method. The phototransistor based on monolayer graphene and SnSe_2_ quantum dots was demonstrated. The photoresponse time was ~0.31 s, and the photoresponsivity was up to 7.5 × 10^6^ mAW^−1^. The n–n heterostructures between monolayer graphene and SnSe_2_ quantum dots enhance the light absorption and the generation of photocarriers. The photocarriers can transfer quickly from SnSe_2_ QDs to graphene, thus improving the photoresponsivity of the device.

## 2. Experiment

**Figure 1:**
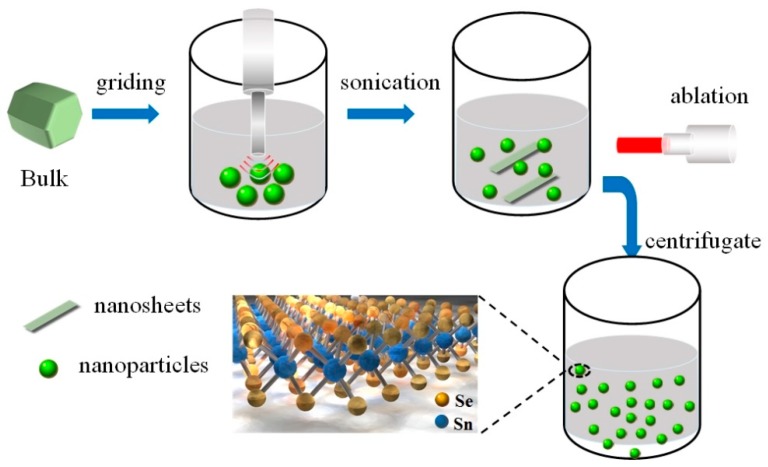
Schematic show of the SnSe_2_ structure and the quantum dot (QD) fabrication process.

SnSe_2_ QDs were fabricated by sonication and the laser ablation process, as shown in Figure 1. The SnSe_2_ bulk was bought from Six Carbon Technology. We put the SnSe_2_ bulk in an agate mortar and manually ground it for 15 min to get SnSe_2_ powders. Then, we dispersed 20 mg of powder in 30 mL of deionized water. The mixture was sonicated with a sonic tip for 2 h at the output power of 650 W in an ice-bath. The power was on for 4 s and off for 2 s. After sonication, the solution was a mixture of SnSe_2_ small particles and flakes. The solution was transferred into a quartz cuvette and irradiated under a 1064 nm pulsed Nd:YAG laser for 10 min (6 ns, 10 Hz). The laser output power was 2.2 W. When the solution was irradiated by the laser pulses, the small particles and flakes absorbed the incident photon energies and formed extreme non-equilibrium conditions (high pressure and temperature) in a short time (~ns). After sustainable irradiation, the particles and nanosheets broke into tiny pieces. Then, the solution was centrifuged for 30 min at a speed of 6000 rpm. After that, the supernatant containing SnSe_2_ QDs was collected.

The morphology of the SnSe_2_ QDs was studied using a high-resolution transmission electron microscope (TEM, FEI Tecnai G2 F30). The structure of the SnSe_2_ QDs was characterized by X-ray diffraction spectroscopy (XRD, Bruker D8 Advance) and the Raman spectra (Horiba Labram HR Evolution). The absorption spectra were measured by a UV-vis spectrometer (Shimadzu UV-1700).

The chemical vapor deposition (CVD)-grown monolayer graphene was wet-transferred onto a p^+^Si/SiO_2_ substrate [14,15]. The thickness of SiO_2_ was 285 nm. The highly doped p-type silicon served as the back-gate electrode. Then, the Cr/Au (10 nm/90 nm) source and drain electrodes were deposited on top of the graphene film by the thermal evaporation method. The channel length and width were 0.2 mm and 2 mm, respectively. The optoelectronic properties were studied using a probe station equipped with a semiconductor parameter analyzer (Keithley 4200). The illumination LED light wavelength was 405 nm.

## 3. Results and Discussion

**Figure 2:**
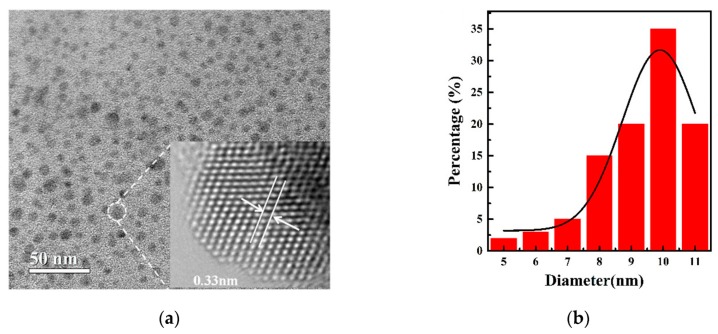
(**a**) TEM image of SnSe_2_ QDs with a centrifugal speed of 6000 rpm. The inset shows the detailed crystal structure of a single QD; (**b**) the size distribution of the SnSe_2_ QDs.

Figure 2a shows the transmission electron microscope (TEM) image of SnSe_2_ QDs as-fabricated. It shows an e- a size distribution in the range of 5–11 nm, and the average size is 9.8 nm, as indicated in Figure 2b. The average size of the QDs comes from the statistical analysis of the sizes of 200 QDs measured from TEM images. A high-resolution TEM image of a single SnSe_2_ QD is shown in the inset of Figure 2a. The lattice spacing is about 0.33 nm, which corresponds to the (1010) planes of a hexagonal-phase SnSe_2_ [16]. The result shows that the SnSe_2_ QDs are crystalline.

**Figure 3:**
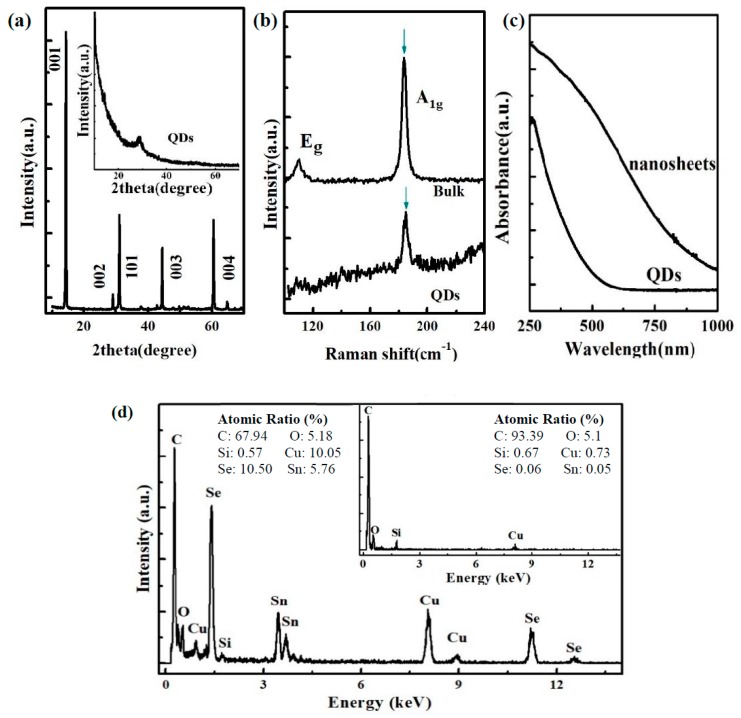
Spectroscopic characterizations. (**a**) XRD pattern of the SnSe_2_ bulk and QDs; (**b**) Raman spectra of the SnSe_2_ bulk and QDs; (**c**) absorption spectra of the SnSe_2_ QDs and nanosheet solutions; (**d**) TEM energy dispersive spectra (TEM-EDS) of the SnSe_2_ QDs. The inset shows the EDS of the TEM substrate without QDs.

Figure 3a shows the XRD patterns of the SnSe_2_ bulk and QDs. The SnSe_2_ bulk has an obvious diffraction peak at *2θ* = 14.4° which corresponds to the (001) faces. In addition, some lower peaks located at *2θ* = 29.1°, *2θ* = 31.2°, *2θ* = 44.3°, and *2θ* = 60.4° are assigned to the (002), (101), (003), and (004) faces. In SnSe_2_ QDs, these diffraction peaks almost disappear except for a tiny peak at *2θ* = 29.1°. After sonication and laser ablation, the SnSe_2_ bulk was cracked into nanoparticles, and there was no constructive interference from the aligned crystal planes [13,17]. The tiny peak at *2θ* = 29.1° corresponds to the (002) face, which may come from the partial restacking of QDs in the process of drying. Figure 3b shows the Raman spectra of the SnSe_2_ bulk and QDs. The incident laser wavelength is 514 nm and the spot size is around 2 μm. For the bulk SnSe_2_, two Raman active vibration modes are observed at 110.3 cm^−1^ and 183.6 cm^−1^, which correspond to the in-plane $E_{g}$ and out-of-plane $A_{1g}$ modes [18]. For the SnSe_2_ QDs, the peak of the $E_{g}$ mode is very weak, but the peak of the $A_{1g}$ mode is observable and has a small blue-shift of ~1 cm^−1^, which may be due to the surface effect and decrease of SnSe_2_ thickness [19].

Figure 3c shows the absorption spectra for SnSe_2_ QDs and SnSe_2_ nanosheets solutions in the range of 250–1000 nm. The absorption band of the SnSe_2_ nanosheets solution is broad, covering regions from the ultraviolet to near-infrared. It is similar to the absorption band reported for the SnSe_2_ powders [20]. For the SnSe_2_ QDs solution, only strong absorption from 250 nm to 420 nm is observed. The bulk SnSe_2_ has an indirect bandgap of 1.0 eV [20]. When the particle size is reduced, the emergence of the quantum confinement effects leads to the discretization of energy levels. As a result, the SnSe_2_ QDs show a larger band gap [21].

Figure 3d shows the TEM energy dispersive spectra (TEM–EDS) of the SnSe_2_ QDs. Tin and selenium can be clearly observed, and their atomic ratios are 5.76% (Sn) and 10.50% (Se), respectively. For comparison, the EDS of the TEM substrate (carbon film-coated copper grid without QDs) is shown in the inset of Figure 3d. The Cu, C, and Si signals come from the TEM grid and sample holder. The O peak arises from the oxygen adsorbed on the surface of the grid. The results show that the QDs are composed of tin and selenium.

**Figure 4:**
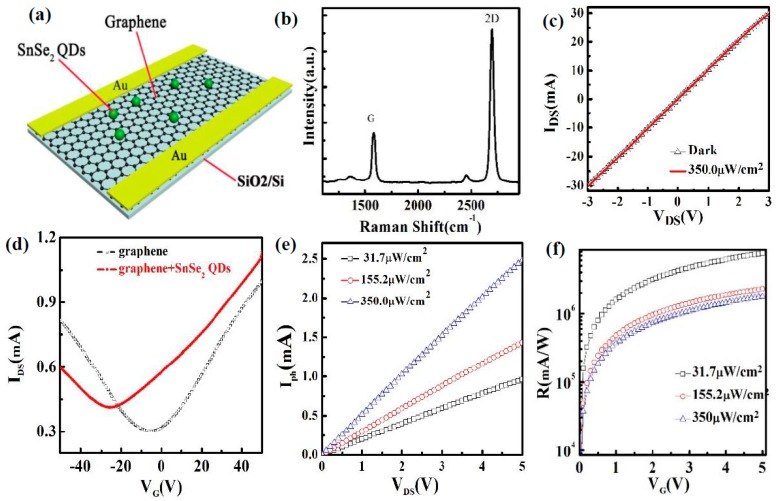
(**a**) Schematic diagram of a graphene photodetector decorated with SnSe_2_ QDs; (**b**) Raman spectra of the pure graphene on a p^+^Si/SiO_2_ substrate; (**c**) the I–V curves for the single-layer graphene phototransistor in the dark and with illumination under zero-gate voltage (V_G_ = 0 V); (**d**) transfer characteristics (I_DS_-V_G_, V_DS_ = 0.5 V) of the phototransistor with and without SnSe_2_ QDs on the graphene film; (**e**) photocurrent and (**f**) responsivity of a SnSe_2_ QD-decorated graphene photodetector as functions of drain voltages at different illumination densities. The illumination wavelength is 405 nm.

Figure 4a schematically shows the photodetector decorated with SnSe_2_ QDs on a p^+^Si/SiO_2_ substrate. The Raman spectra of the pure graphene on a p^+^Si/SiO_2_ substrate is shown in Figure 4b. Two Raman peaks at 1582 cm^−1^ (G line) and 2698 cm^−1^ (2D line) are observed. The ratio of the integrated intensities of the G line and 2D line is ~0.25. The peak at 1350 cm^−1^ (D line) in the spectra is very weak, indicating that the graphene is a monolayer with good quality. The I–V curves for the monolayer graphene phototransistor in the dark and with illumination under zero gate voltage (V_G_ = 0 V) are shown in Figure 4c. The illumination density is 350 μW/cm^2^. As shown in the figure, there is no change between the current in the dark and under illumination, indicating that the photoresponse of pure graphene is negligible.

Figure 4d shows the transfer curves (I_DS_-V_G_, V_DS_ = 0.5 V) of the device with and without SnSe_2_ QDs in which the light is absent. The transfer curve of the device without SnSe_2_ QDs exhibits a typical V-shape. The field-effect mobilities are ~230 cm^2^V^−1^s^−1^ for electrons and ~220 cm^2^V^−1^s^−1^ for holes. The negative, neutral charge point (about −5 V) of single-layer graphene is observed in Figure 4d, indicating an electron dominated conduction in the graphene. The same behavior was also observed by Sun et al. [10]. Graphene is very sensitive to the surroundings. The defects in the SiO_2_ substrate, residues from processing and handling, charged impurities, and substrate surface roughness can cause the shift of the neutral charge point [22]. The SnSe_2_ QDs solution was dropped on the top of graphene film and heated at 40 °C for 30 min in a glove box filled with N_2_ gas. The transfer curve of the photodetector with SnSe_2_ QDs becomes asymmetric, and the Dirac point converts to a negative gate voltage (about −22 V). The shift indicates that the SnSe_2_ QDs are n-type semiconductors, which are the same type as the bulk SnSe_2_ [20]. The electron and hole mobilities decrease to ~160 cm^2^V^−1^s^−1^ and ~130 cm^2^V^−1^s^−1^, respectively.

In order to study the optoelectronic properties of the device, we measured the photocurrents at different illumination densities with zero gate voltage (V_G_ = 0 V). Figure 4e shows the relationship between the photocurrent (I**_Ph_** = I**_Light_** − I**_Dark_**) and the applied drain voltages. I**_Light_** is the drain current under illumination, and I**_Dark_** is the drain current without illumination. The photocurrent increases while increasing the illumination density. Figure 4f represents the responsivity (R = $I_{\mathrm{ph}}/\left({\mathrm{WLE}}_{e}\right)$) of the photodetector as functions of drain voltages at different illumination densities. The responsivity decreases while increasing the illumination density, which is consistent with the reported UV-detectors [23]. The maximum responsivity of the device is about 7.5 × 10^6^ mAW^−1^ (V_DS_ = 5 V) at an incident power density of 31.7 μW/cm^2^, which is higher than that reported in graphene-based UV phototransistors [24,25,26].

**Figure 5:**
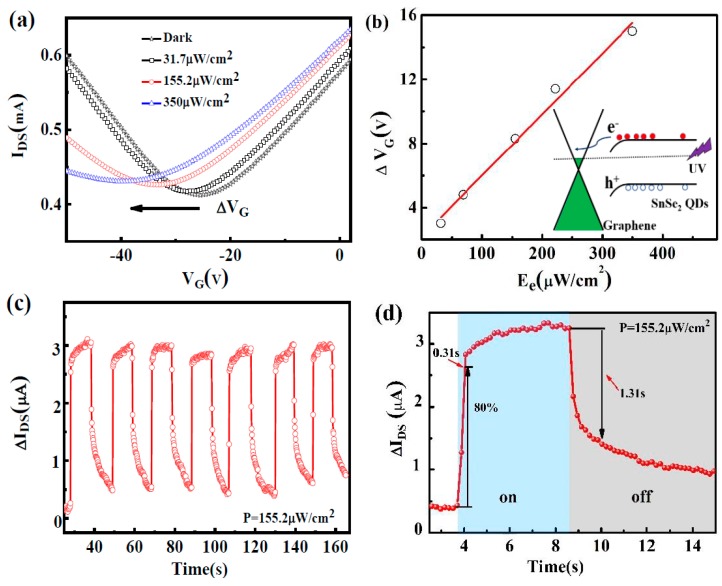
(**a**) Transfer characteristics of a graphene photodetector decorated with SnSe_2_ quantum dots at different illumination densities (wavelength: 405 nm, V_DS_ = 0.5 V); (**b**) horizontal shift of transfer curves as functions of illumination densities. The inset shows the charge transfer between SnSe_2_ QDs and graphene; (**c**) current response to on/off light illumination for several cycles; (**d**) photocurrent response time of the device. (V_DS_ = 0.05 V, illumination density: 155.2 μW/cm ^2^).

Figure 5a shows the transfer curves of the photodetector at different illumination densities. The Dirac point of the device shifts to a lower negative gate voltage while increasing illumination density. The shift of the transfer curves (ΔV_G_) is plotted as a function of illumination densities in Figure 5b. The shift of the transfer curve (ΔV_G_) changes linearly with the light illumination density (Ee), indicating that the photo-induced carrier density in SnSe_2_ QDs increases with increasing illumination density. This illumination density-dependent shift does not appear in the pure graphene phototransistor. The existence of SnSe_2_ QDs leads to this photoresponse behavior. As shown in Figure 5a, the electron mobility in the SnSe_2_ QD-decorated device is higher than that of holes at different illumination densities. The photo-induced electron-hole pairs are separated at the interface between SnSe_2_ QDs and monolayer graphene. The SnSe_2_ QDs/graphene heterojunction facilitates the injection of photo-generated electrons from SnSe_2_ QDs into the graphene, leading a local n-doping in the graphene channel. Since the transfer rate of holes is lower that of electrons, net positive charges remain in the SnSe_2_ QDs. Then, a lower negative gate voltage is required to obtain the charge neutral point (Dirac point) in the detector. A similar process was reported in a p-doped graphene/PbS QDs phototransistor by Sun et al. [9].

Figure 5c shows the current response to on/off light illumination and Figure 5d shows the photocurrent response time of the device (V_G_ = 0 V, V_DS_ = 0.05 V, illumination density: 155.2 μW/cm^2^). The photocurrent increases with time when the illumination is on and decreases with time when the illumination is off. As shown in Figure 5d, the photocurrent increases to 80% with a response time of 0.31 s, which is faster than that reported in graphene devices [9,10,24,26,27]. The response time includes charge generation time, charge transfer time in heterojunctions, and charge collection time. In our experiment, the measured graphene charge mobility is smaller than the value for perfect graphene (up to 200,000 cm^2^V^−1^s^−1^), which may be due to the defects induced in the graphene film while transferring to the substrate, and the response time can be improved by optimizing the graphene transfer process. When the light is turned out, the photocurrent decreases to 20% with a time of 1.31 s.

**Figure 6:**
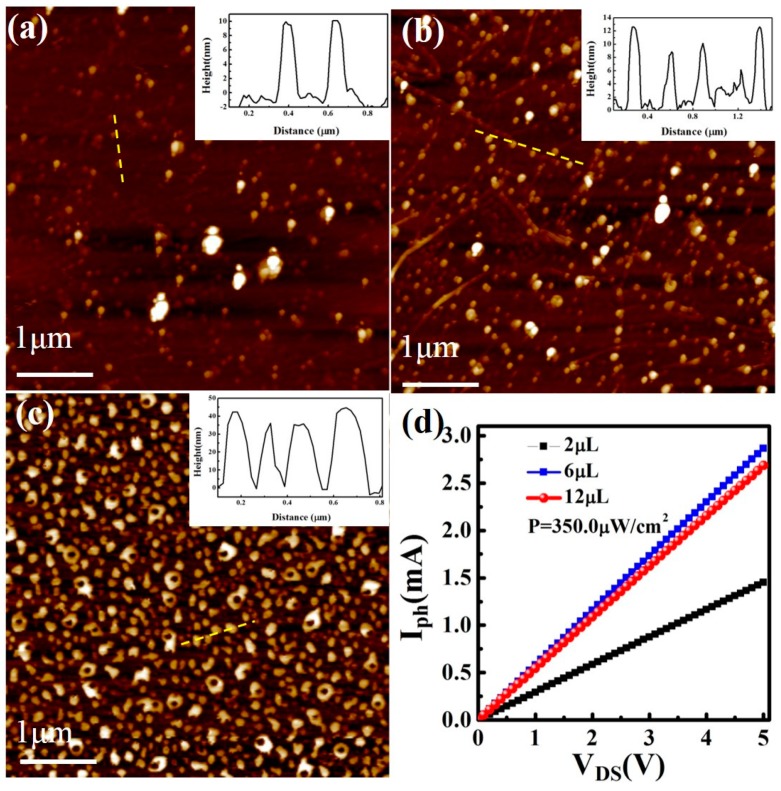
The AFM images of SnSe_2_ QDs with different densities (**a**) 2 μL, (**b**) 6 μL, and (**c**) 12 μL. The insets show their height profiles. (**d**) The photocurrents with different SnSe_2_ QDs densities at the irradiation density of 350.0 μW/cm^2^.

The photocurrent of the detector is influenced by the SnSe_2_ QDs density. We have measured the AFM pictures and photocurrents for detectors with different SnSe_2_ QDs densities. As shown in Figure 6, the photocurrent increases with an increase of the SnSe_2_ QDs density under the same irradiation density (illumination density: 350 μW/cm^2^). When the SnSe_2_ QDs thickness is larger than 40 nm, the photocurrent tends to decrease, which may be due to the decrease of the charge transfer between the QDs layers.

## 4. Conclusions

In summary, uniformly distributed SnSe_2_ quantum dots were synthesized at room temperature using a facile and environment-friendly method. The UV-detector based on monolayer graphene and SnSe_2_ quantum dots was demonstrated. The device showed fast photoresponse time of ~0.31 s, and its photoresponsivity was up to 7.5 × 10^6^ mAW^−1^. The n–n heterostructures between monolayer graphene and SnSe_2_ QDs improved the light absorption and the transportation of photocarriers, which have promising applications in optoelectronic devices.
